# Supplementary material for: Revealing real-time 3D in vivo pathogen dynamics in plants by label-free optical coherence tomography
Source: Nat Commun. 2024 Sep 27;15:8353. doi: 10.1038/s41467-024-52594-x (PMC11437094; doi:10.1038/s41467-024-52594-x)
Supplement: Supplementary file 1 — Supplementary Information [file 41467_2024_52594_MOESM1_ESM.pdf]

Revealing real-time 3D in vivo pathogen dynamics in plants by label-free optical coherence tomography

Jos de Wit[1], Sebastian Tonn[2], Mon-Ray Shao[2], Guido Van den Ackerveken[2], and Jeroen Kalkman[1,*]

[1]Department of Imaging Physics, Delft University of Technology, Lorentzweg 1, 2628 CJ Delft, The Netherlands
[2]Translational Plant Biology, Department of Biology, Utrecht University, Padualaan 8, 3584 CH Utrecht, The Netherlands
[*]j.kalkman@tudelft.nl

1 Dynamic OCT contrast analysis and optimization

For quantitative pathogen imaging with dynamic OCT contrast optimization is pivotal. The dynamic OCT (dOCT) contrast optimization for *Bremia lactucae* consisted of three steps. First, the optimal frequency range of the dynamic OCT signal for the three RGB colors was chosen for the identification of biologically active material, see section 1.1. Second, for the generation of high-contrast RGB images the optimal top and bottom values of the (logarithmic) intensity values were determined, see section 1.2. Third, the binary contrast for *B. lactucae* segmentation based on color value combinations was optimized, see section 1.3.

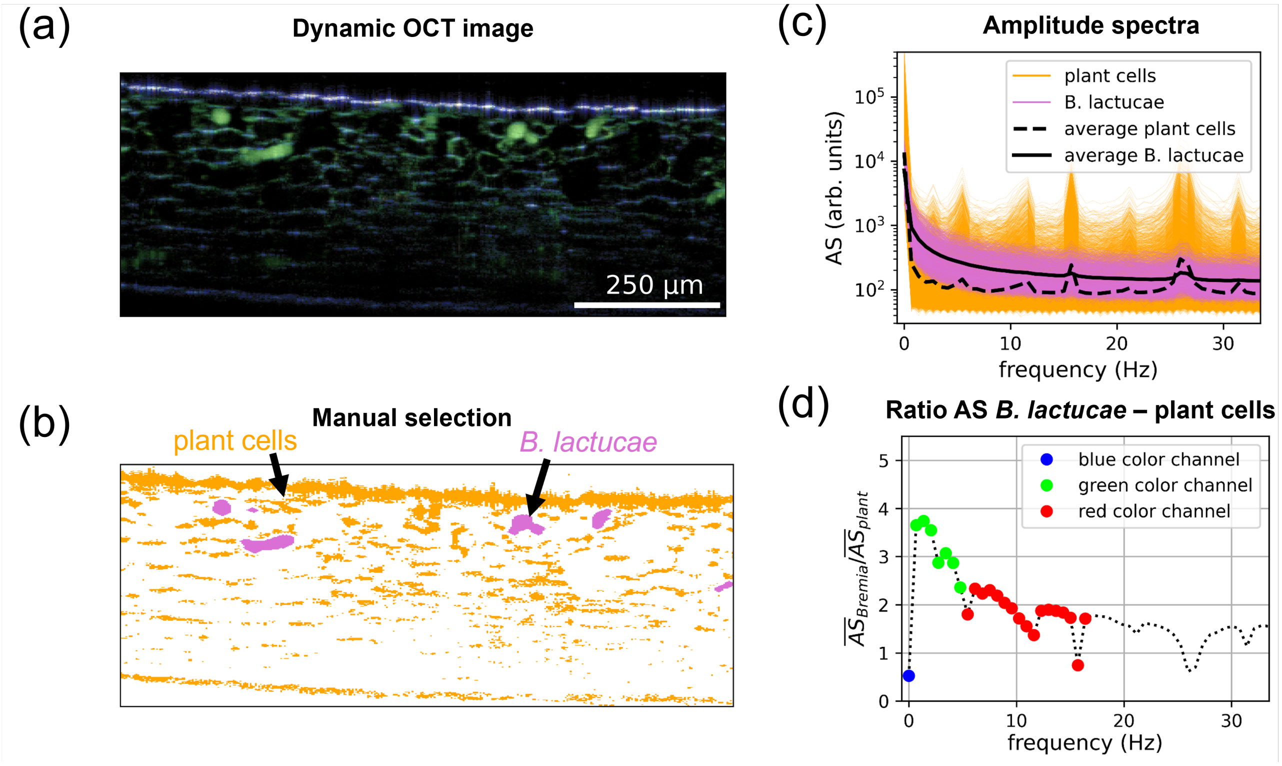

Supplementary Figure 1. Analysis of the contrast between plant cells and *Bremia lactucae*. (a) The dynamic OCT image that was used for the analysis. (b) The segmentation of *B. lactucae* and plant cells. *B. lactucae* was manually segmented, and plant cells were selected above an OCT intensity threshold. (c) The amplitude spectra of *B. lactucae* and plant cell pixels. (d) The ratio between the average AS of *B. lactucae* and plant cells.

1.1 Color band frequency range selection

For the analysis and optimization of the frequency ranges for each color, 2048 B-scans were obtained with 14.6 ms repetition rate from a sample with *B. lactucae*. This scan was then divided into 39 overlapping sets of 100 B-scans for which the amplitude spectrum (AS) was calculated per pixel. For every pixel, the 39 AS were averaged. Then a dynamic OCT B-scan was obtained from these average AS using empirically determined frequency bands obtained by visually inspecting the resulting contrast. The thus obtained dOCT images had sufficient contrast to clearly distinguish the *B. lactucae*. The dOCT image from the average AS (here with the optimized frequency bands, reprocessed after optimization) is shown in Suppl. Fig. 1(a).

From this image, the *B. lactucae* hyphae were manually segmented as *B. lactucae* pixels. Plant cells were identified from being above a threshold in the average conventional OCT signal, while the *B. lactucae* pixels and their neighbor pixels were excluded. This resulted in the segmentation shown in Suppl. Fig. 1(b).

Supplementary Figure 1(c) shows the AS of *B. lactucae* and plant cells. Plant cells are highly static and thus show a high value at 0 Hz, and much lower values at higher frequency. For increasing frequency, the AS quickly drops to a stable, horizontal plateau which is the flat frequency spectrum corresponding to the shot noise detector response. The orange lines show the AS of individual pixels, but the trend is clearer for the average AS. *B. lactucae* starts with a lower value at 0 Hz, but, due to its active nature, the decay is a lot slower and the AS remains high for the medium frequencies just after the 0 Hz sampling point.

Note that the amplitude at 0 Hz represents the average signal over time and is proportional to the average scattering strength. The relatively high refractive index contrast of the plant cells generates a high 0 Hz signal, but also the *B. lactucae* hyphae have a considerable mean scattering strength. The significant difference is in the ratio between the signal at 0 Hz and other frequencies, which indicates how much of the mean scattering strength fluctuates due to tissue dynamics. The dOCT images encode this ratio through the mixture of colors, while the brightness of the image is still related to the scattering strength.

From the amplitude spectra, it is clear that the difference between plant cells and *B. lactucae* is most significant for the ratio between the AS from the first non-zero frequency to 4.8 Hz, and the static signal at 0 Hz, indicating that *B. lactucae* shows most activity at the used resolution at time scales between 0.2 and 1.4 s. The small peaks around 16 and 27 Hz are probably caused by fixed-pattern noise and those are not present in all pixels, thus they cannot serve as features to distinguish between plant cells and *B. lactucae*.

The ratio between the average AS of *B. lactucae* and plant cells, as shown in Suppl. Fig. 1(d) supports the finding that *B. lactucae* has increased power in the low-frequency range. Based on the ratio of AS we chose the blue color for the dOCT signal at the 0 Hz sampling point, the green color band for 0.7 to 4.8 Hz, and the red color band for higher frequencies at 5.5 to 16.4 Hz. The frequency sampling points are indicated in (d) with points that have the color of the frequency band they belong to. Frequencies above 16.4 Hz mainly contain noise, and therefore did not contribute to meaningful contrast. Excluding them from the dOCT signal also allowed for reducing the amount of required frames per set to 50, thus increasing the volume that could be imaged within the same time. Shifting the edge between the green and red color band with one or two sampling points did not show any significant change in the dOCT images, thus the contrast is relatively robust, and further optimization was unnecessary.

1.2 Dynamic range optimization of the color bands

After obtaining the most optimal frequency bands, they need to be efficiently displayed. OCT images are usually log-compressed for sufficient contrast between different scattering levels, with the higher limit around the maximum scattering strength and the lower limit around the noise floor. Here, we have three different color channels that have their own noise floor and maximum amplitude. Thus the upper and lower limit of their dynamic range has to be determined for each color channel, such that the colors are balanced for obtaining high-quality images[1].

Supplementary Figure 2(a) shows the detailed process of obtaining dOCT images with optimized dynamic range from the amplitude spectra. We used the same value for all volumes within the experiment to ensure that the relative colors mean the same for all images.

The lower limit of the dynamic range per color was determined from the histograms of the average AS calculated on a 1024-bin grid within the noise level region, which excluded around 5% of the voxels with the highest amplitude[2,3]. The histograms are dominated by the noise signal due to the open structure of the leaf, the significant image portion outside the leaf, and the larger variation of the amplitude of scattered signal[2]. The histograms were determined for each volume and then summed to get an overall histogram per color channel, Supplementary Figure 2(b). A parabolic fit of the peak in a ±30-bin region resulted in an accurate estimate of the peak location, which was used as the lower edge of the dynamic range. This procedure ensures that all signals above the noise floor are included while reducing background noise. The small differences in peak positions between individual volumes are less than 2% of the total dynamic range and may result from small variations in the reference arm intensity.

The upper edge of the dynamic range was obtained by averaging the maximum value of each of the volumes. Choosing the peak value as the maximum ensures a large dynamic range that includes the large majority of the high-intensity signal,

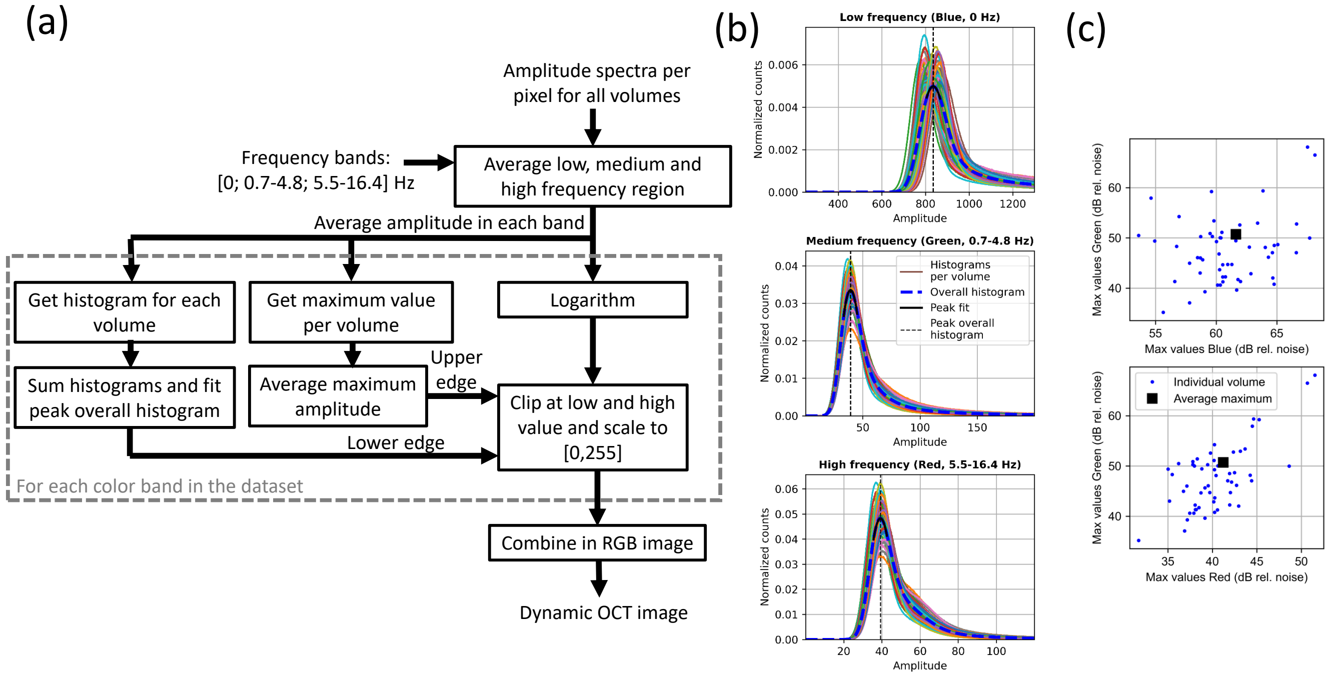

Supplementary Figure 2. (a) Detailed processing pipeline from amplitude spectra to the dOCT image, including determination of bottom and top limit of each color range. (b) The histograms for each color channel and each volume, together with the overall histogram and the fit of the peak. (c) The maximum values of green vs blue (top) or green versus red (bottom), with the average maximum taken as the upper dynamic range limit for all volumes.

but clips some highest intensity signals thereby avoiding too dark images. For individual volumes (e.g., the examples with other plant-pathosystems), the maximum intensity within the same volume was taken as the upper edge. Supplementary Figure 2(c) shows the maximum values for each volume and the average. Note that the average of the maxima was taken before log-compressing. It is thus on the upper side of the scattered points.

Note that the chosen limits could differ for different OCT setups (with different noise levels) and experiments (with different maximum intensities). However, images from the same setup are sufficiently consistent to interpret dOCT images. For quantitative comparison, the dynamic range limits were chosen equal for all volumes within an experiment.

1.3 Monochromatic contrast optimization for segmentation

After determination of the dynamic range for the RGB channels, they are combined to create a monochrome image. For optimal contrast between *B. lactucae* and plant cells, a weighted combination of the RGB colors was taken to determine the monochromatic signal C, i.e.,

$$C = \alpha R + \beta G + \gamma B, \tag{1}$$

where R, G, and B are the color values between the upper and lower range and α, β, and γ are the weighting coefficients.

The monochromatic contrast C was optimized using an exploration of the parameter space of $[\alpha, \beta = 1, \gamma]$, using manually segmented B-scans taken from Gaussian filtered volumes as outlined in the Parameter combination analysis in Supplementary Figure 3(a). Second, a threshold was chosen based on this analysis and a few iterations were done to optimize the threshold of the signal C (Empirical threshold optimization).

In the first step, the relevant values of α, γ with $\beta = 1$ were explored using manually segmented *B. lactucae* in 6 B-scans from Gaussian smoothed dynamic OCT volumes (with the same sigma as for the segmentation). The B-scans from all three genotypes were selected on the presence of pathogen hyphae, and the hyphae were segmented manually in ImageJ. This gave 8493 pathogen voxels with RGB values and 1395507 non-pathogen voxels with RGB values. For these voxels, the contrast was calculated for values $-1 \leq \alpha \leq 2$ and $-3 \leq \gamma \leq 0$ on a 64×64 grid. For each contrast, different thresholds were evaluated that would include 50% up to 98% of the pathogen pixels. Then the number of non-pathogen voxels above the threshold was obtained, i.e., the false positive voxels. This number should be as low as possible to avoid detection of non-pathogenic structures. Also, if non-pathogenic structures are present, they should be sufficiently sparsely scattered such that most of them can be removed by applying a filter that removes small objects.

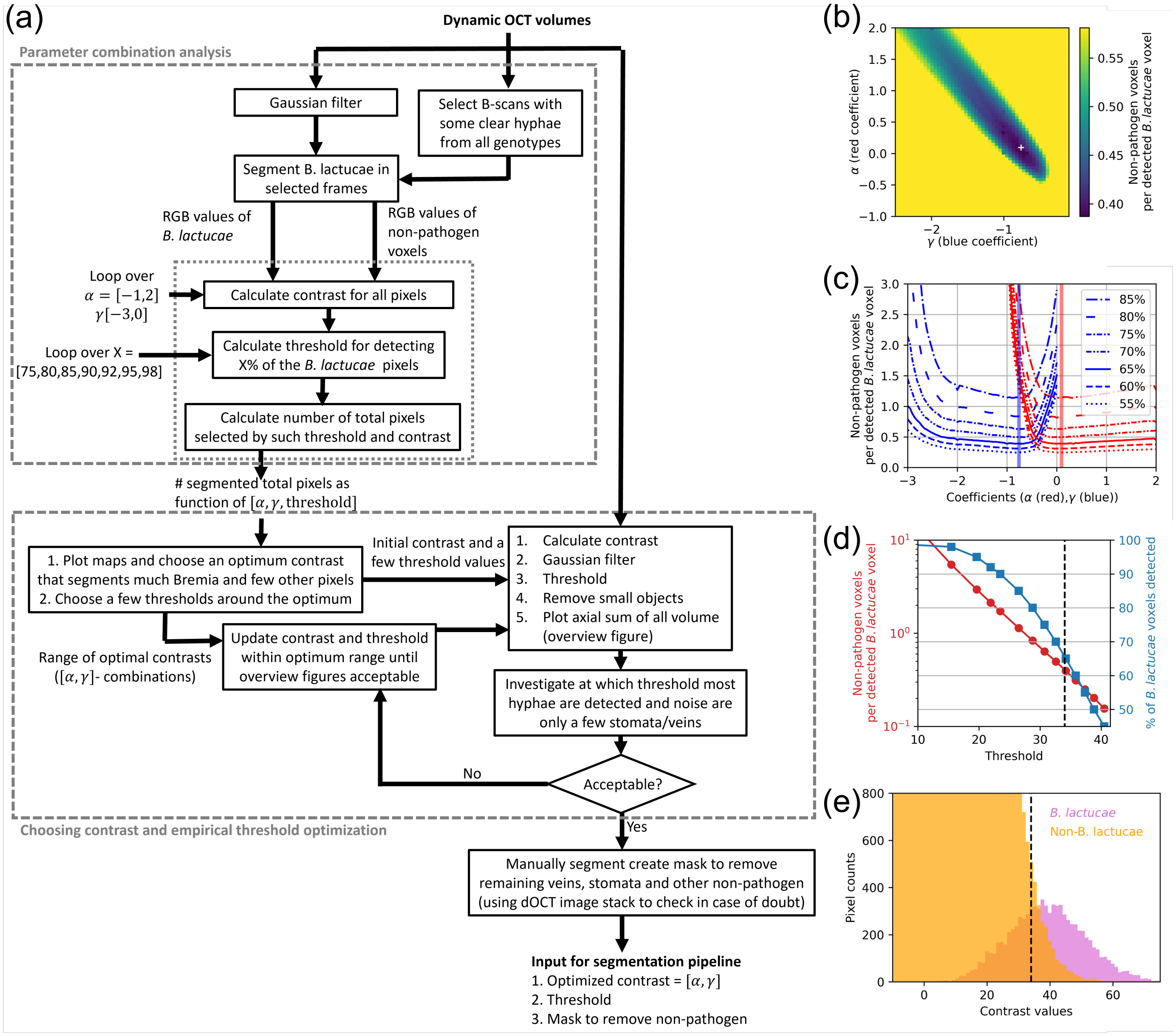

Supplementary Figure 3. Monochromatic contrast optimization pipeline. (a) The flowchart of the contrast optimization. (b) The number of non-pathogen voxels per pathogen voxel plotted for different combinations of α and γ. The threshold was chosen such that 65% of the pathogen voxels were included. (c) The minimum intensity projection of (b) along both axes for thresholds that include different percentages of the pathogen voxels (55-85%). (d) The number of non-pathogen voxels above the threshold and the percentage of detected pathogen voxels plotted against the threshold at the chosen optimum contrast. (e) Histogram of the optimum contrast values for the non-pathogen and *B. lactucae* voxels. The vertical dashed lines in (d-e) indicate the chosen threshold.

Supplementary Figure 3(b) plots the number of false positive voxels per detected pathogen voxel for different combinations of α and γ, for a threshold for which 65% of the pathogen voxels are detected. The figure shows an elongated optimum area with a negative correlation between α and γ, showing that a more negative value for γ requires a more positive value for α to avoid a threshold close to 0 (which would include many noise pixels without any signal). For different percentages of detected pathogen pixels (giving a different threshold), the slope of this pattern varies slightly but the optimum regions largely overlap. Based on this graph, we chose a $\alpha = 0.095$ and $\gamma = -0.762$, as the most optimal choice, indicated by the white cross.

Supplementary Figure 3(c) shows the number of detected non-pathogen voxels for different percentages of detected pathogen voxels, plotted against α and γ, with the optimal γ and α respectively. This shows that γ can take values between -2.2 and -0.6 with a slight optimum around -0.8. Outside this region, the separation between non-pathogen and pathogen quickly

worsens, as shown by the increasing number of non-pathogen voxels. For α, any value above 0 seems acceptable, as long as it is combined with the right γ, although there is a slight preference for values around or slightly above 0. The vertical lines show the chosen contrast for α and γ, which are close to the optimal for all percentages of detected pathogen voxels.

With the selected α, β, and γ the threshold is first optimized using the manually segmented B-scans by calculating the number of wrongly detected plant voxels per detected pathogen voxel and the percentage of detected pathogen voxels, which is plotted against the threshold, see Supplementary Figure 3(d). The detected number of non-pathogen voxels per pathogen voxel decreases exponentially, while the percentage of detected pathogen voxels decreases much slower. The most optimum threshold was aimed to detect sufficiently few non-pathogen voxels, such that most voxels are sparsely distributed and can be removed with the filter that removes small objects. For this reason, we accepted a loss of 20-40 % of the pathogen voxels and explored thresholds between 30 and 39 in the second stage of empirical threshold optimization.

In the second threshold optimization step, the optimized contrast of $\alpha = 0.095$, $\beta = 1$, and $\gamma = -0.762$ was tested for all volumes in the dataset with thresholds between 30 and 39, and the axial sum of segmented voxels was plotted for all volumes, as in Supplementary Figure 4. This approach quickly shows at different thresholds whether most hyphae are obtained and how many stomata, veins, and some other active cell parts remain. Based on these results, we chose a threshold where the number of stomata, veins, and a few other active cell parts was small such that they could be manually segmented out on a 2D maximum intensity projection image. We found that a threshold of 34 gave satisfactory results. Supplementary Figure 3(e) shows the histograms of non-pathogen and pathogen voxels for the 6 segmented B-scans that were used for the contrast optimization with the the optimum threshold as vertical line.

Using the results shown in Supplementary Figure 3(d), it could be estimated that with the optimum threshold, around 65% of the pathogen is detected. There is thus an unavoidable bias in the detected volume to the low side since otherwise too many non-pathogen voxels will be included. In practice, the number of included non-pathogen voxels per detected pathogen voxel will be higher than the numbers in the graphs, because the B-scans that were used for the analysis had more than average pathogen presence. Still, the second step showed that most non-pathogenic voxels were filtered out by the filter that removes small objects.

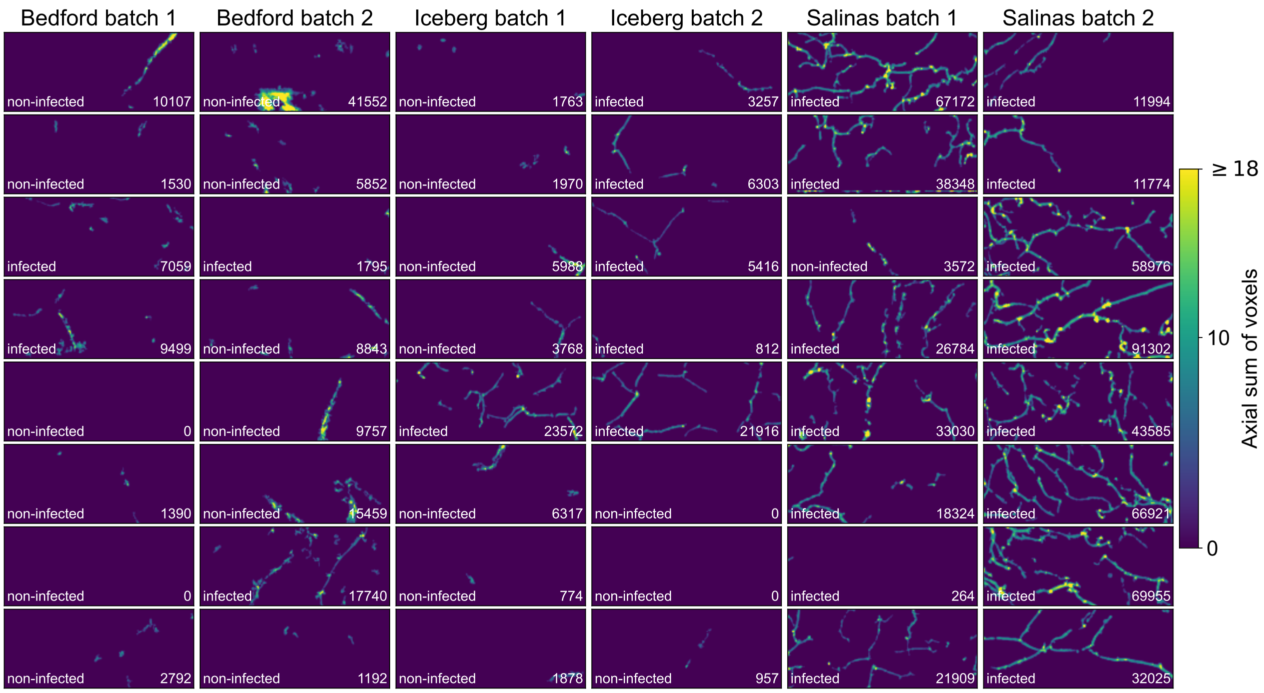

Supplementary Figure 4. The axial sum of the segmented *Bremia lactucae* voxels without using a mask. Veins, some stomata and some artefacts and active epidermal cells are visible in the images, which are segmented out to obtain a clean segmentation. The bottom left shows whether any pathogen is detected (based on manual inspection of dOCT images), while the bottom right shows the number of segmented voxels without mask.

2 Threshold analysis

The threshold has a significant influence on the absolute quantification of the *Bremia lactucae* hyphae volume and length. Figure 5(a) shows the box plots as in Fig. 2(c) of the manuscript for a range of threshold values around the optimal value of 34. For most thresholds, the comparison between the genotypes shows the same pattern. The removal of stomata, veins, and some active cells by manual segmentation was done only for structures that were visible with the threshold of 34, thus the box plots for thresholds below this value can be polluted with non-pathogen voxels. This is especially clear for the thresholds of 30, where Bedford gives a higher signal than Iceberg. The plant cells of Bedford, especially around veins, were more active in the medium frequency range than Iceberg and Salinas cells, thus giving a larger pollution with non-pathogen voxels.

Besides the effect of including non-pathogen voxels at low thresholds, the threshold also influences where the edge of hyphae are chosen (mainly impacting the absolute volume) and whether some hyphae with a weaker signal are included (impacting both volume and length). This effect is shown in Supplementary Figure 5(b-c), where the volume and length decrease in a close to linear way with an increasing threshold. The ratio between the volume at the highest and lowest threshold is 0.06, 0.35, and 0.53 for Bedford, Iceberg and Salinas respectively, Bedford being lower because of the higher pollution at a threshold of 30. For the length, the ratios are 0.06, 0.43, and 0.68, which is higher as the length suffers less from hyphae that become thinner.

Despite the variation in the absolute volume and length the relative changes are similar. To demonstrate this we compare the p-values for the different thresholds, see Supplementary Figure 5(d). We see that for thresholds above 33 the P-values are almost constant, which shows that the comparison of different genotypes is robust against threshold variation. The differences below a threshold of 33, are mainly the result of pollution by non-pathogen voxels of veins and stomata as only those that appear above a threshold of 34 are removed. Overall, the quantification of resistance is quite robust with respect to the choice of the threshold value.

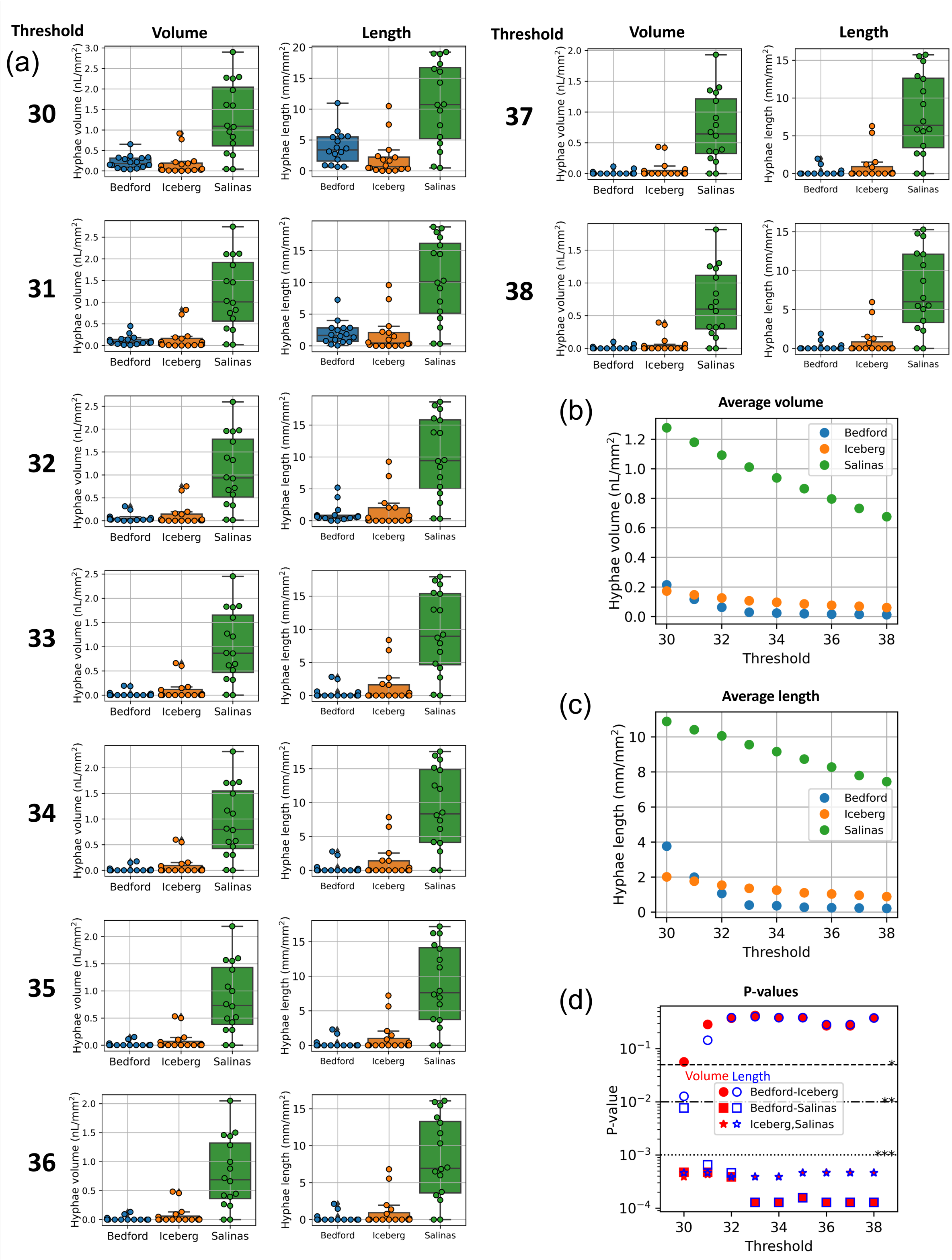

Supplementary Figure 5. Results for quantitative data with different thresholds. (a) Volume and hyphae length box plots for thresholds between 30 and 38. For the box plots, the middle line represents the median value, the lower and upper quartile lines represent the 25th and 75th percentile, and the whiskers show the maximum and minimum values of data points that are not qualified as outliers. Outliers are visible as points outside the whiskers. (b-c) The average hyphal volume (b) and length (c) for each variety plotted against the threshold. (d) the p-values with the double-sided Mann-Whitney U test with Benjamini Hochberg p-value adjustment for the different combinations. Red is volume and blue is length. Source data for all figures are provided in a Source Data file.

3 Overview images experiment 1

Supplementary Figure 6 shows the maximum intensity projections of the Gaussian filtered optimized contrast ($\alpha = 0.095$, $\beta = 1$ and $\gamma = -0.762$) of all the samples of experiment 1 (Fig. 2 of the manuscript). Supplementary Figure 4 shows the axial sum of the segmented *B. lactucae* voxels at the optimum threshold of 34 without applying the mask, showing veins, some stomata, and also a few artefacts due to a saturated detector. Segmenting out these non-pathogen structures and using that as mask yields clean segmentation of hyphae, whose axial sum images are shown in Supplementary Figure 7. In each image of Supplementary Figures 4 and 7 it is indicated in the bottom left whether the volume was infected (based on manual inspection of the dOCT image stack) and at the bottom right, how many voxels were segmented out as hyphae.

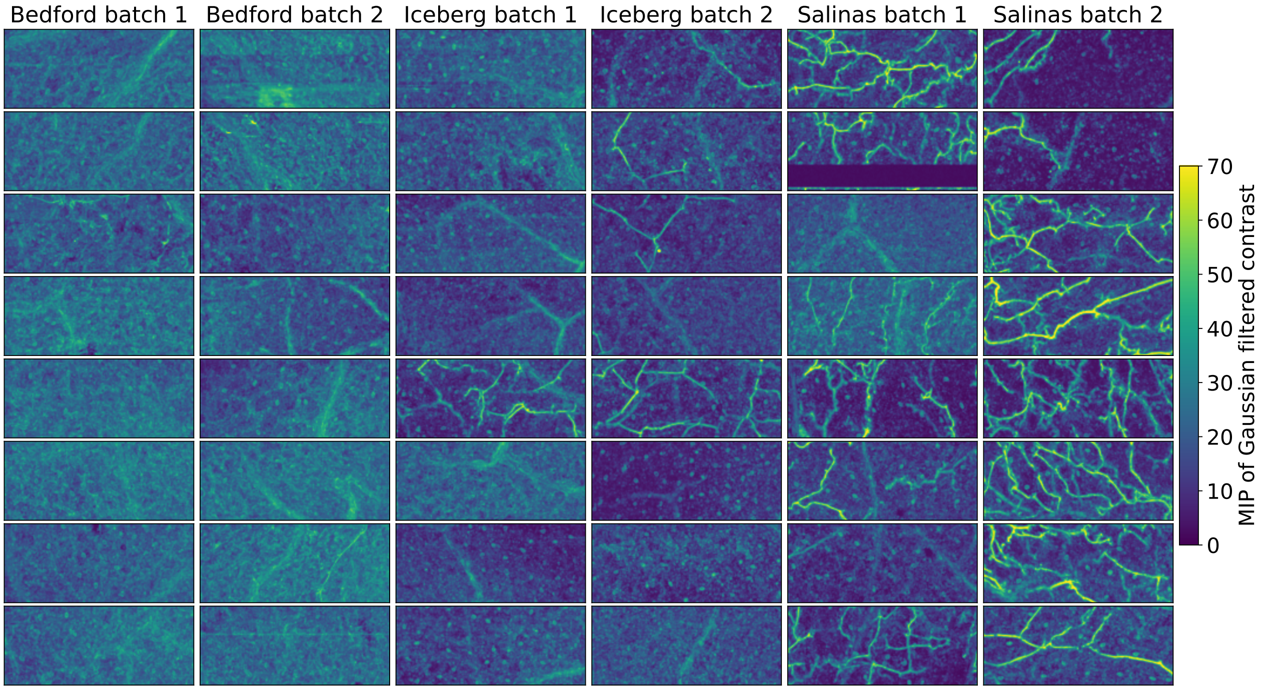

Supplementary Figure 6. Maximum intensity projection (MIP) images of all images of experiment 1 after calculating *Bremia lactucae* contrast and Gaussian filtering. The images were taken in two badges (over two days) as indicated above the columns. The second image from Salinas batch 1 contains one-third where the data acquisition failed. To keep a fair comparison, the hyphae volume and length of this volume are multiplied by 3/2.

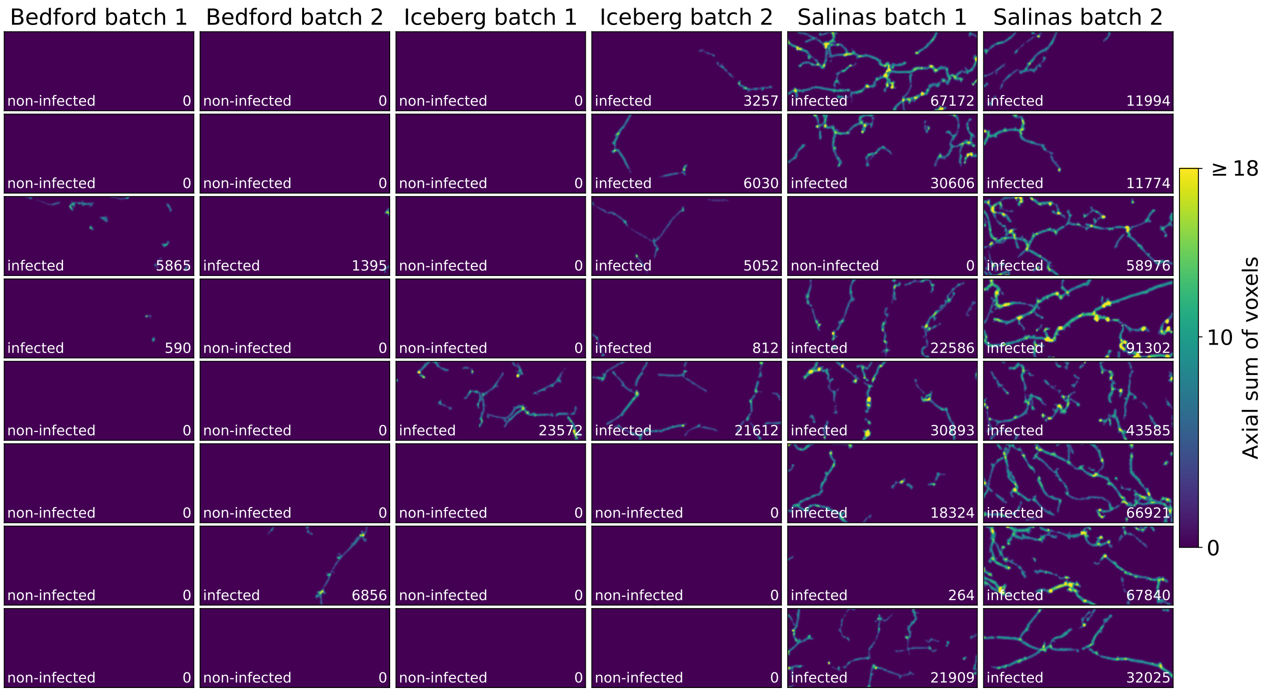

Supplementary Figure 7. Axial sum of the segmented *Bremia lactucae* voxels. The left bottom of each image indicates whether the volume is infected, and the bottom right shows the number of segmented voxels. Note that some infected volumes do not have all hyphae segmented, due to the trade-off between robustness against noise and accuracy of the segmentation.

4 Statistical analysis

Supplementary Table 1. Statistical values of the samples in the quantitative experiment. Grouped by (dynamic OCT-based) hyphal volume, (dynamic OCT-based) hyphal length, and qPCR, this table gives the number of samples, the mean, standard deviation, minimum, and maximum of the dataset of the cultivar.

Cultivar	Bedford	Iceberg	Salinas
**N samples volume dynamic OCT**	16	16	16
**Mean volume (nL/mm^2)**	0.023	0.096	0.938
**Standard deviation volume (nl/mm^2)**	0.0531	0.1869	0.6694
**Maximum volume (nL/mm^2)**	0.174	0.598	2.316
**Minimum volume (nL/mm^2)**	0.0	0.0	0.0
**N samples length dynamic OCT**	16	16	16
**Mean length (mm/mm^2)**	0.358	1.253	9.159
**Standard deviation length (mm/mm^2)**	0.8297	2.3496	5.8185
**Maximum length (mm/mm^2)**	2.778	7.84	17.543
**Minimum length (mm/mm^2)**	0.0	0.0	0.0
**N samples qPCR**	5	5	5
**Mean qPCR (Bl_{mtDNA}/Ls_{gDNA})**	0.691	4.44	43.4
**Standard deviation qPCR (Bl_{mtDNA}/Ls_{gDNA})**	0.533	3.58	20.9
**Maximum qPCR (Bl_{mtDNA}/Ls_{gDNA})**	1.70	9.98	69.7
**Minimum qPCR (Bl_{mtDNA}/Ls_{gDNA})**	0.126	0.455	22.9

Supplementary Table 2. Parameters of the statistical tests for the quantitative experiment. Grouped by (dynamic OCT-based) hyphal volume, (dynamic OCT-based) hyphal length, and qPCR, this table gives the uncorrected p-value, effect size, confidence interval, and corrected p-value. For the volume and length, P-values are calculated with the double-sided Mann-Whitney U test, and p-value correction was performed using the Benjamini-Hochberg method. For the qPCR data, differences were evaluated using the two-sided Welch's t-tests assuming unequal variance.

Cultivar combination	Bedford-Iceberg	Bedford-Salinas	Iceberg-Salinas
**P-value uncorrected volume**	0.371	9.51e-06	8.03e-05
**Effect size (nL/mm^2)**	-2.39e-05	-0.784	-0.784
**Confidence interval (nL/mm^2)**	[-2.06e-2, 9.44e-06]	[-1.48, -0.465]	[-1.165, -0.382]
**P-value corrected volume**	0.384	1.42e-04	3.83e-04
**P-value uncorrected length**	0.371	9.51e-06	8.03e-05
**Effect size (mm/mm^2)**	-9.44e-05	-8.14	-7.74
**Confidence interval (mm/mm^2)**	[-0.389, 8.08e-05]	[-12.90, -4.17]	[-12.52, -4.04]
**P-value corrected length**	0.384	1.42e-04	3.83e-04
**P-value uncorrected qPCR**	0.104	0.015	0.0193
**Effect size (Bl_{mtDNA}/Ls_{gDNA})**	-3.75	42.7	38.9
**Confidence interval (Bl_{mtDNA}/Ls_{gDNA})**	[-8.70, 1.19]	[13.7, 71.7]	[10.12, 67.8]
**P-value corrected qPCR**	0.104	0.029	0.029

5 Imaging other pathogens and other crops

The general applicability of dOCT is exemplified by three additional examples of imaging in other plant species infected by different downy mildews or plant-parasitic nematodes. Supplementary Figure 8 shows horizontal plane cross-sections of OCT and dOCT images for three different plant species and pathogens or parasites. First, *Arabidopsis thaliana* leaf tissue infected with downy mildew *Hyaloperonospora arabidopsis* shows little contrast in the normal OCT image, but bright green contrast in the dOCT image. With the same contrast that was used for *B. lactucae* segmentation (i.e., $\alpha = 0.095$, $\beta = 1$ and $\gamma = -0.762$), followed by Gaussian filtering and a maximum intensity projection (MIP), the hyphae in the whole imaged leaf section become clearly visible. Second, radish (*Raphanus sativus subsp. sativus*) infected with downy mildew (*Hyaloperonospora brassicae f. sp. raphani*) shows less strong contrast in color between the plant cells and hyphae, but the structure of the plant cells and hyphae still enables identification of the hyphae in the dOCT image. The MIP of the optimized contrast for *B. lactucae* shows the hyphae again with a clear contrast. Third, root-knot nematodes (*Meloidogyne incognita*) inside pepper (*Capsicum annuum*) roots can also be visualized by dOCT imaging. Though much of the structure can be seen already in the normal OCT image, the dOCT image enables better distinction between nematode eggs and the gelatinous matrix that is holding them and also improves the contrast between plant cells and the nematode. For the root-knot nematode, the roots were washed but no infiltration was applied.

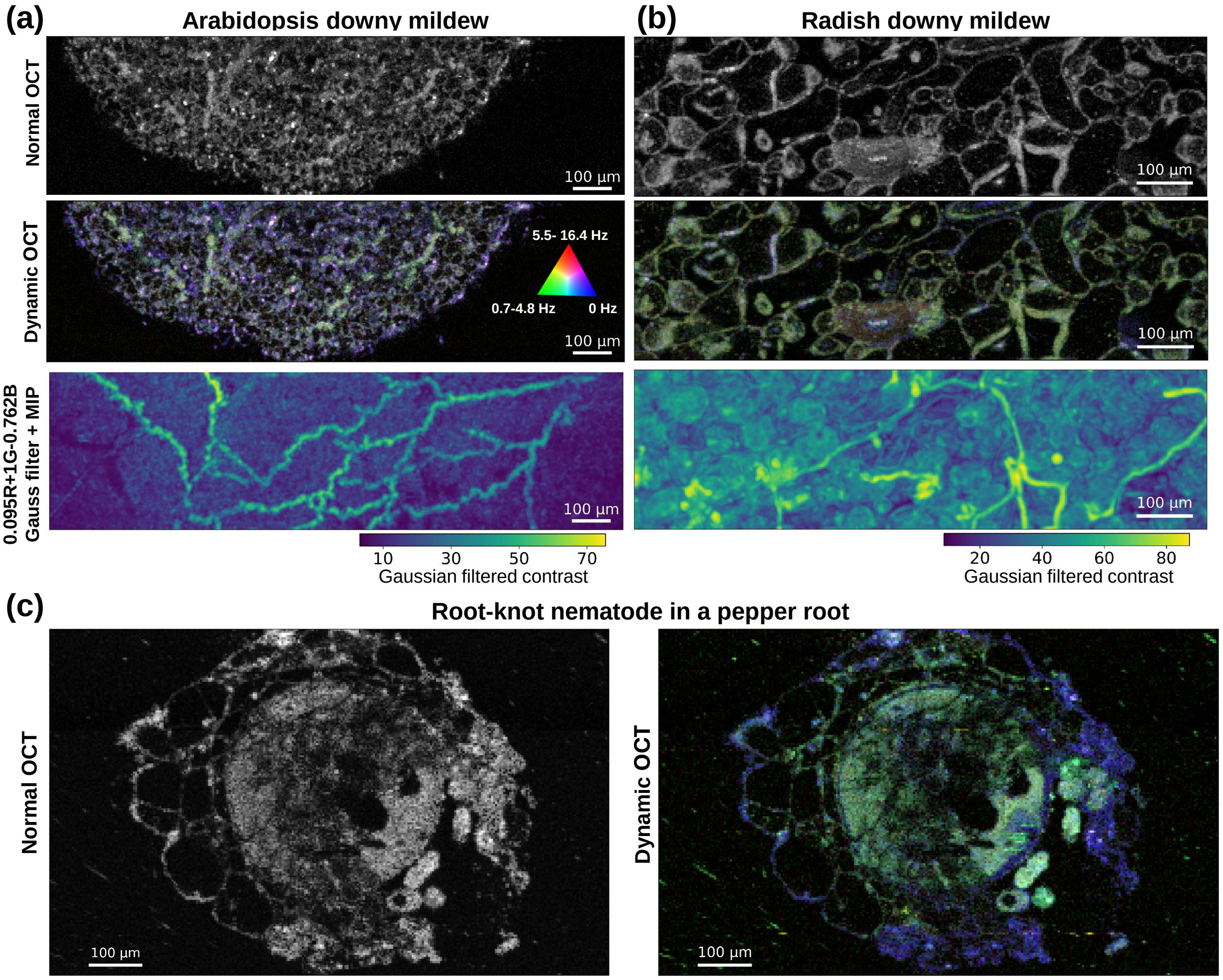

Supplementary Figure 8. Horizontal plane cross sections from normal and dynamic OCT (dOCT) 3D images results for (a) *Arabidopsis* infected with *Hyaloperonospora arabidopsis*, (b) Radish downy mildew (*Hyaloperonospora brassicae f. sp. raphani* in a susceptible radish accession) and (c) Root-knot nematode (*Meloidogyne incognita*) in pepper (*Capsicum annuum*) root. The figures compare normal OCT (averaged over 50 frames) with dOCT. For (a-b) the maximum intensity projection (MIP) over the full axial range of Gaussian filtered *B. lactucae* contrast (i.e., $\alpha = 0.095$, $\beta = 1$ and $\gamma = -0.762$) is also displayed, clearly showing the downy mildew hyphae.

6 Overview images experiment 2

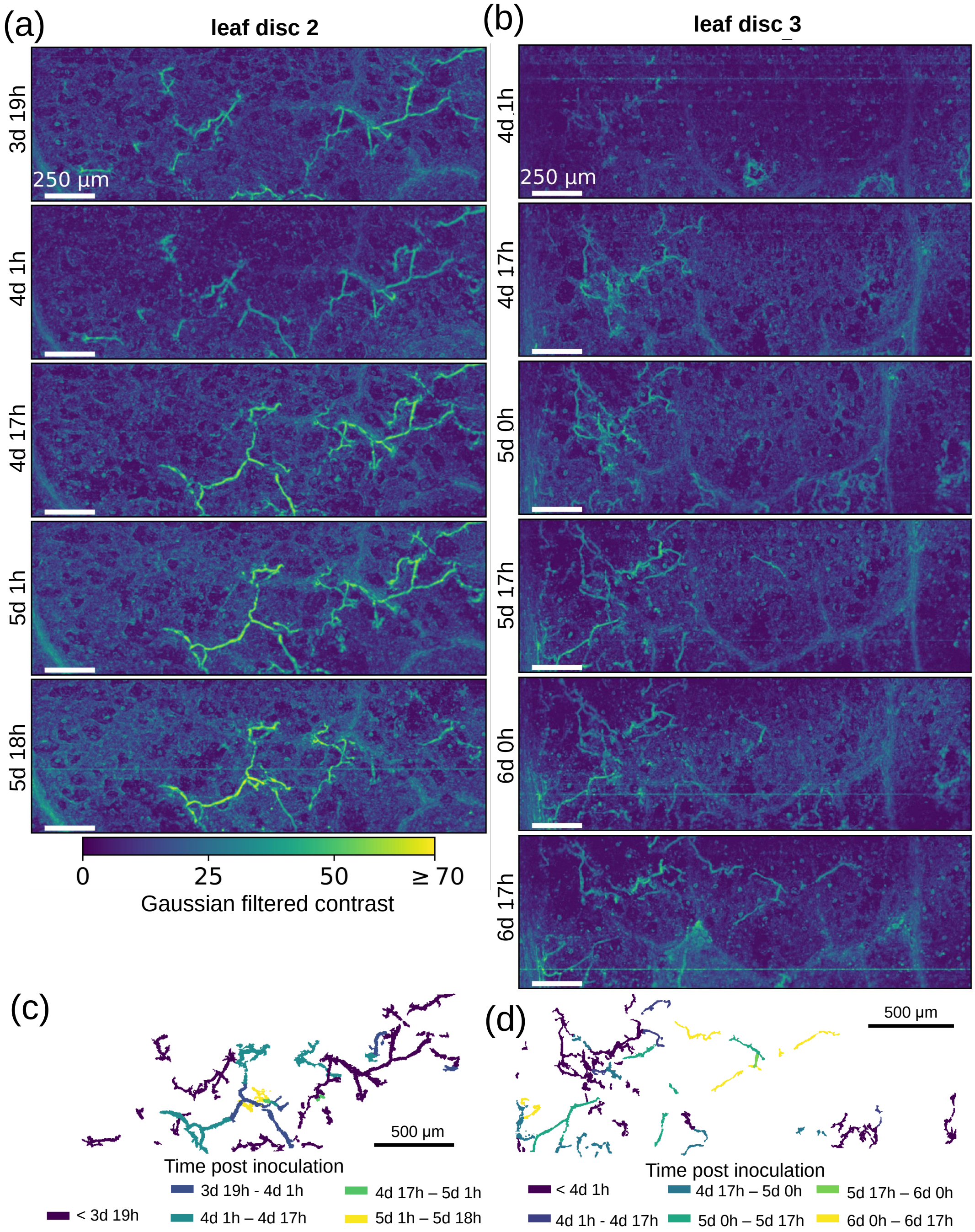

Supplementary Figure 9. Time-lapse overview figure of maximum intensity projections of Gaussian filtered *Bremia lactucae* contrast for (a) disc 2 and (b) disc 3 in Figure 3(c) of the manuscript. For disc 2 in the left half, many hyphae disappear from the *B. lactucae* image due to faded activity, while new hyphae grow in that direction. For disc 3, it was more challenging to determine all times of origin as the imaged area in the second image was slightly shifted and the focus depth was not always the same. This is most likely the reason for the bottom right part at 5d 17h not showing clear hyphae, while the next image shows active hyphae again. Still, by assessing the 3D dOCT image, a good estimation could be made of when the hyphae appeared, which is summarized in (c) for disc 2 and (d) for disc 3.

References

1. Münter, M. *et al.* Dynamic contrast in scanning microscopic OCT. *Opt. Lett.* **45**, 4766–4769 (2020).

2. Steiner, P., Kowal, J. H., Považay, B., Meier, C. & Sznitman, R. Automatic estimation of noise parameters in Fourier-domain optical coherence tomography cross sectional images using statistical information. *Appl. Opt.* **54**, 3650–3657 (2015).

3. De Wit, J., Angelopoulos, K., Kalkman, J. & Glentis, G.-O. Fast and accurate spectral-estimation axial super-resolution optical coherence tomography. *Opt. Express* **29**, 39946–39966 (2021).
