# Supplementary material for: Revealing real-time 3D in vivo pathogen dynamics in plants by label-free optical coherence tomography
Source: Nat Commun. 2024 Sep 27;15:8353. doi: 10.1038/s41467-024-52594-x (PMC11437094; doi:10.1038/s41467-024-52594-x)
Supplement: Supplementary file 3 — Description of Additional Supplementary Files [file 41467_2024_52594_MOESM3_ESM.pdf]

<u>Description of Additional Supplementary Files</u>

Supplementary Movie 1:

Part I: shows the dynamics of a germinating B. lactucae spore imaged with brightfield microscopy and OCT.

The left image shows the time-lapse of a germinating spore made with a brightfield microscope with differential interference contrast. This sequence shows the motion of the sub-cellular content over time.

The middle image shows the time lapse of an OCT image of a spore on agarose gel illustrating the fluctuating speckle in time. This image is the same as what can be seen on the screen during real-time normal OCT imaging. Note that the bright dots in the agarose (bottom half) do not fluctuate (only the lower-intensity noise pixels), while in the spore the high-intensity speckle fluctuates.

The right image shows the dynamic OCT image of the same spore as the middle panel, obtained just after the middle image sequence. These images show the motion as discussed in Figure 1(c) of the manuscript.

Part II: shows dynamic OCT imaging of a downy mildew-infected lettuce leaf.

The left shows the normal OCT image (vertical cross-section) in time, as could be seen while doing real-time OCT imaging. The fluctuating speckle is clearly visible in this time-lapse, which can serve as a real-time feedback mechanism during the exploration of the leaf sample for infection. The right shows the corresponding dynamic OCT image, where the hyphae are visualized in bright green due to the medium frequency speckle fluctuation. A small bright dot at the right half could have been missed in the normal OCT image but is clearly visible in the dynamic OCT image. Also, a stoma is visible on the right side. Several hyphae and the stoma are indicated with an arrow.

Part III shows real-time OCT imaging of a large vertical cross-section of downy mildew-infected lettuce leaf tissue. The leaf sample is moved in the in-plane direction by manually turning the knot of a translation stage. With the real-time feedback of the OCT imaging, and the visually

striking speckle motion, areas with a high density of B. lactucae hyphae can be found and the path of hyphae can be followed. This part of the visualization is mainly intended to indicate how real time OCT imaging could already help to investigate infection, after which dynamic OCT imaging can be used to capture the infection with clear contrast.

Supplementary Movie 2: This visualization shows a volume rendering of one of the infected volumes of experiment 1 (the quantification of downy mildew infection for different lettuce varieties) in the different stages of segmentation of Fig. 2(b). This volume did not have many veins and stomata above the threshold, so the step of removing them did not result in much change.
