# Supplementary material for: Revealing real-time 3D in vivo pathogen dynamics in plants by label-free optical coherence tomography
Source: Nat Commun. 2024 Sep 27;15:8353. doi: 10.1038/s41467-024-52594-x (PMC11437094; doi:10.1038/s41467-024-52594-x)
Supplement: Supplementary file 6 — Reporting Summary [file 41467_2024_52594_MOESM6_ESM.pdf]

nature portfolio

Corresponding author(s): Jeroen Kalkman

Last updated by author(s): Aug 21, 2024

Reporting Summary

Nature Portfolio wishes to improve the reproducibility of the work that we publish. This form provides structure for consistency and transparency in reporting. For further information on Nature Portfolio policies, see our Editorial Policies and the Editorial Policy Checklist.

Statistics

For all statistical analyses, confirm that the following items are present in the figure legend, table legend, main text, or Methods section.

n/a	Confirmed	
☐	☒	The exact sample size (*n*) for each experimental group/condition, given as a discrete number and unit of measurement
☐	☒	A statement on whether measurements were taken from distinct samples or whether the same sample was measured repeatedly
☐	☒	The statistical test(s) used AND whether they are one- or two-sided<br>*Only common tests should be described solely by name; describe more complex techniques in the Methods section.*
☐	☒	A description of all covariates tested
☐	☒	A description of any assumptions or corrections, such as tests of normality and adjustment for multiple comparisons
☐	☒	A full description of the statistical parameters including central tendency (e.g. means) or other basic estimates (e.g. regression coefficient) AND variation (e.g. standard deviation) or associated estimates of uncertainty (e.g. confidence intervals)
☐	☒	For null hypothesis testing, the test statistic (e.g. *F*, *t*, *r*) with confidence intervals, effect sizes, degrees of freedom and *P* value noted<br>*Give P values as exact values whenever suitable.*
☒	☐	For Bayesian analysis, information on the choice of priors and Markov chain Monte Carlo settings
☒	☐	For hierarchical and complex designs, identification of the appropriate level for tests and full reporting of outcomes
☒	☐	Estimates of effect sizes (e.g. Cohen's *d*, Pearson's *r*), indicating how they were calculated

Our web collection on statistics for biologists contains articles on many of the points above.

Software and code

Policy information about availability of computer code

Data collection	ThorImage 5.4.1 for OCT imaging and the attached C++ DLL with custom Python 3.7 code running in Anaconda Spyder.
Data analysis	Most data analysis was done in Python 3.7, running in the Anaconda Spyder console. This includes the segmentation described in Figure 2 and supplement. Relevant scripts and data to make the figures are shared in a Zenodo repository.<br>For investigating and 3D visualization of the 3D dynamic OCT image stacks, ImageJ was used. For point cloud visualization and segmentation for the growth of the downy mildew over time we used CloudCompare (V2.13.alpha), as described in the methods. Statistical tests with corrections were done using R4.4.0.

For manuscripts utilizing custom algorithms or software that are central to the research but not yet described in published literature, software must be made available to editors and reviewers. We strongly encourage code deposition in a community repository (e.g. GitHub). See the Nature Portfolio guidelines for submitting code & software for further information.

Data

Policy information about availability of data

All manuscripts must include a data availability statement. This statement should provide the following information, where applicable:

- Accession codes, unique identifiers, or web links for publicly available datasets
- A description of any restrictions on data availability
- For clinical datasets or third party data, please ensure that the statement adheres to our policy

All analysis code and some representative data examples are available at https://zenodo.org/doi/10.5281/zenodo.11428245

Research involving human participants, their data, or biological material

Policy information about studies with human participants or human data. See also policy information about sex, gender (identity/presentation), and sexual orientation and race, ethnicity and racism.

Reporting on sex and gender	No data related to sex or gender was collected
Reporting on race, ethnicity, or other socially relevant groupings	no data on race, ethnicity or socially relevant grouping was collected or used.
Population characteristics	No human research participants were involved.
Recruitment	n.a.
Ethics oversight	n.a.

Note that full information on the approval of the study protocol must also be provided in the manuscript.

Field-specific reporting

Please select the one below that is the best fit for your research. If you are not sure, read the appropriate sections before making your selection.

☒ Life sciences ☐ Behavioural & social sciences ☐ Ecological, evolutionary & environmental sciences

For a reference copy of the document with all sections, see nature.com/documents/nr-reporting-summary-flat.pdf

Life sciences study design

All studies must disclose on these points even when the disclosure is negative.

Sample size	No statistical analysis was done to determine sample sizes. For the quantitative resistance experiment we chose a sample size (8 leaf discs with 2 areas imaged per leaf disc) that was technically feasible and in the range of sample sizes used in previous downy mildew disease screens in the Translational Plant Biology Group at Utrecht University. In these experiments, that also included the lettuce cultivars used in this study, sample sizes above 6 proved to be sufficient to reveal quantitative differences in downy mildew resistance, based on quantification of pathogen sporulation or pathogen DNA.<br>For the qPCR analysis in Fig.2 we used 5 whole leaves per genotype, based on experience of getting a good illustration of the trend of susceptibility.<br>For the time lapse experiment, we imaged 10 samples at multiple time points. The number was limited by the availability of the imaging equipment and our ambition to image each sample twice every day. The three samples presented in the manuscript were selected based on consistent image quality over sufficient time points and presence of downy mildew to demonstrate the ability of imaging over the course of a few days.
Data exclusions	For the time lapse experiment we selected 3 out of 10 measured samples based on image quality, presence of downy mildew infection, and sufficient successful measurement points. We excluded samples that turned out to not contain any downy mildew hyphae or samples with limited number of high quality images e.g. due to wrong focus or shifted field of view at single time points. As the goal of this experiment was to illustrate the ability to follow in-vivo pathogen growth over time, and we did not draw statistically significant conclusions about the plant-pathogen system, we judged that it is acceptable to select three samples with sufficient quality.
Replication	Imaging downy mildew colonization inside leaf tissue was replicated many times in independent experiments, as also shown in the data presented in the manuscript. The level of contrast varied as also shown in the data in the manuscript and supplementary materials, but was sufficiently consistent to apply segmentation and time-lapse comparison.<br><br>Imaging of alive spores with dynamic OCT was repeated >10 times and each time multiple spores were in view. Imaging alive spores with DIC contrast widefield microscopy was done at least 3 times with multiple spores in view. Imaging dead spores was done at least 2 times with >10 individual spores inside the field of view. Verification with Trypan blue staining after dynamic OCT imaging was done for 3 samples, of which one was not infected (and showed no hyphae in both the dynamic OCT as the Trypan blue image). The measurements on Arabidopsis and radish with downy mildew infection, and the nematode were only done once as a example, but with real time OCT an area of interest was

determined as discussed at randomization.

We also observed the quantitative differences in downy mildew colonization between lettuce cultivars Bedford, Iceberg and Salinas in independent experiments with alternative quantification methods (e.g. quantification of pathogen sporulation). The intermediate resistance level of Iceberg and the strong resistance of Bedford to B. lactucae race Bl: 33EU is also documented in literature (e.g. DOI: 10.1094/PHYTO-08-20-0367-R) and by the International Bremia Evaluation Board (IBEB) (https://worldseed.org/our-work/disease-resistance/other-initiatives/ibeb/).

Randomization	For the quantitative resistance experiment, leaf discs punched from the third true leaf of indicidual plants were randomly placed in a 3 x 4 grid in a petri dish, 4 leaf discs per each of the 3 genotypes. Two petri dishes were prepared in this way. After randomization, leaf discs were spray inoculated with Bremia lactucae.<br>For imaging, 2 areas per leaf disc were randomly chosen, but such that they were at least 1 mm away from the leaf disc edge (to avoid edge effects) and sufficiently flat to obtain a good image.<br><br>For the time lapse images, we searched for a location where we could see hyphae (based on the temporal fluctuations of the signal in real-time B-scan OCT imaging) and chose that area for imaging, to have a greater opportunity to see growth of the pathogen. These areas are thus not randomly chosen. When no clear infection was spotted, a random area was chosen.<br><br>For the Arabidopsis and radish with downy mildew infection, and the nematode in pepper root, the imaging location was actively searched for using real-time OCT imaging and the naked eye (nematode).
Blinding	No blinding was done, but for the quantitative study the area was chosen without first imaging and the infections were not visible by eye. In the analysis and segmentation, the steps were as much as possible automated or done based on a global data picture.

Reporting for specific materials, systems and methods

We require information from authors about some types of materials, experimental systems and methods used in many studies. Here, indicate whether each material, system or method listed is relevant to your study. If you are not sure if a list item applies to your research, read the appropriate section before selecting a response.

Materials & experimental systems

n/a	Involved in the study	
☒	☐	Antibodies
☒	☐	Eukaryotic cell lines
☒	☐	Palaeontology and archaeology
☒	☐	Animals and other organisms
☒	☐	Clinical data
☒	☐	Dual use research of concern
☐	☒	Plants

Methods

n/a	Involved in the study	
☒	☐	ChIP-seq
☒	☐	Flow cytometry
☒	☐	MRI-based neuroimaging

Dual use research of concern

Policy information about dual use research of concern

Hazards

Could the accidental, deliberate or reckless misuse of agents or technologies generated in the work, or the application of information presented in the manuscript, pose a threat to:

No	Yes	
☒	☐	Public health
☒	☐	National security
☒	☐	Crops and/or livestock
☒	☐	Ecosystems
☒	☐	Any other significant area

Experiments of concern

Does the work involve any of these experiments of concern:

No	Yes	
☒	☐	Demonstrate how to render a vaccine ineffective
☒	☐	Confer resistance to therapeutically useful antibiotics or antiviral agents
☒	☐	Enhance the virulence of a pathogen or render a nonpathogen virulent
☒	☐	Increase transmissibility of a pathogen
☒	☐	Alter the host range of a pathogen
☒	☐	Enable evasion of diagnostic/detection modalities
☒	☐	Enable the weaponization of a biological agent or toxin
☒	☐	Any other potentially harmful combination of experiments and agents

Plants

Seed stocks	Radish plant infected by radish downy mildew, pepper plant infected by root knot nematode and seeds of lettuce cultivars Bedford, Iceberg and Salinas were provided by Rijk Zwaan B.V. (De Lier, the Netherlands). Arabidopsis thaliana Col-0 plant infected by Hyaloperonospora arabidopsidis was obtained from the Plant-Microbe Interactions group, Utrecht University.
Novel plant genotypes	n.a.
Authentication	The plants were labeled when they were sown. Additional authentication was based on visual inspection of the plants and their known traits (e.g. seed color, leaf color, growth type).
